# Integrated analysis of long non-coding RNAs and mRNAs reveals the regulatory network of maize seedling root responding to salt stress

**DOI:** 10.1186/s12864-021-08286-7

**Published:** 2022-01-13

**Authors:** Peng Liu, Yinchao Zhang, Chaoying Zou, Cong Yang, Guangtang Pan, Langlang Ma, Yaou Shen

**Affiliations:** State Key Laboratory of Crop Gene Exploration and Utilization in Southwest China, Maize Research Institute, Sichuan Agricultural University, Chengdu, 611130 P. R. China

**Keywords:** Maize, Salt stress, lncRNA, TFs, Protein-coding transcript, WGCNA

## Abstract

**Background:**

Long non-coding RNAs (lncRNAs) play important roles in response to abiotic stresses in plants, by acting as cis- or trans-acting regulators of protein-coding genes. As a widely cultivated crop worldwide, maize is sensitive to salt stress particularly at the seedling stage. However, it is unclear how the expressions of protein-coding genes are affected by non-coding RNAs in maize responding to salt tolerance.

**Results:**

The whole transcriptome sequencing was employed to investigate the differential lncRNAs and target transcripts responding to salt stress between two maize inbred lines with contrasting salt tolerance. We developed a flexible, user-friendly, and modular RNA analysis workflow, which facilitated the identification of lncRNAs and novel mRNAs from whole transcriptome data. Using the workflow, 12,817 lncRNAs and 8,320 novel mRNAs in maize seedling roots were identified and characterized. A total of 742 lncRNAs and 7,835 mRNAs were identified as salt stress-responsive transcripts. Moreover, we obtained 41 cis- and 81 trans-target mRNA for 88 of the lncRNAs. Among these target transcripts, 11 belonged to 7 transcription factor (TF) families including *bHLH*, *C2H2*, *Hap3/NF-YB*, *HAS*, *MYB*, *WD40*, and *WRKY*. The above 8,577 salt stress-responsive transcripts were further classified into 28 modules by weighted gene co-expression network analysis. In the salt-tolerant module, we constructed an interaction network containing 79 nodes and 3081 edges, which included 5 lncRNAs, 18 TFs and 56 functional transcripts (FTs). As a trans-acting regulator, the lncRNA *MSTRG.8888.1* affected the expressions of some salt tolerance-relative FTs, including protein-serine/threonine phosphatase 2C and galactinol synthase* 1*, by regulating the expression of the *bHLH* TF.

**Conclusions:**

The contrasting genetic backgrounds of the two inbred lines generated considerable variations in the expression abundance of lncRNAs and protein-coding transcripts. In the co-expression networks responding to salt stress, some TFs were targeted by the lncRNAs, which further regulated the salt tolerance-related functional transcripts. We constructed a regulatory pathway of maize seedlings to salt stress, which was mediated by the hub lncRNA *MSTRG.8888.1* and participated by the *bHLH* TF and its downstream target transcripts. Future work will be focused on the functional revelation of the regulatory pathway.

**Supplementary Information:**

The online version contains supplementary material available at 10.1186/s12864-021-08286-7.

## Background

Globally, over 831 million hectares of the land have been influenced by salinity (http://www.fao.org), which reduces the water availability, inhibits the growth and development, and causes the decreased yield in crops [1]. The main effects of salt stress in plant are as follows: (1) High ambient concentrations of salt increase the cell water potential, change the cell osmotic pressure, hence cause the loss of cell water, and result in physiological drought [2]. (2) The accumulated ions break cell ion balance and thus cause ion toxicity, due to the nutrient imbalance in the cytosol. (3) Salt stress disturbs reactive oxygen species (ROS) and induces oxidative stress [2–4]. Maize (*Zea mays L.*) is a widely cultivated crop worldwide, and it is sensitive to salt stress particularly at the seedling stage [5]. Understanding how maize responds to salt stress could help to develop salt-tolerant maize lines for maize breeding. In maize, large numbers of protein-coding genes involved in salt stress have been reported in previous studies. Overexpression of the *Suaeda salsa* Na^+^/H^+^ antiporter gene (*SsNHX1*) in maize enhances the salt tolerance of the transgenic maize [6]. *ZmSnRK2.11* is a potential negative regulator involved in maize salt stress, which is up-regulated by high salinity. Overexpression of *ZmSnRK2.11* in *Arabidopsis* caused the salt sensitivity phenotypes, including increased rate of water loss, reduced relative water content, and delayed stoma closure [7]. The high-affinity potassium transporter gene (*HKT1*) affects K^+^ and Na^+^ transports in roots and shoots, regulates K^+^/Na^+^ homeostasis, and thus improves the tolerance to Na^+^ stress in maize [8]. The *HAK* family ion transporter *ZmHAK4* confers the shoot Na^+^ exclusion and salt tolerance in maize by retrieving Na^+^ from xylem sap [9]. In addition, some transcript factors (TFs) are also associated with salt stress response in maize. For instance, *ZmMYB3R* positively regulates maize tolerance to salt stress via an ABA-dependent pathway [10]. Some other TF families including *HSF*, *NAC*, *WRKY*, and *bZIP* have been reported to participate the response to salt stress [11–15]. Furthermore, several signaling-related genes and plant hormones-related genes were proven to correlate with maize salt tolerance [16–19]. However, the molecular regulatory networks of these genes have not been fully elucidated. Especially, it is still obscure how the expression of protein-coding genes is affected by non-coding RNA in maize [20].Long non-coding RNA (lncRNA) is a type of non-coding RNA that has ≥ 200 nucleotides in length. Based on their genomic localizations relative to protein-coding genes, lncRNAs are mainly classified into long intergenic ncRNAs (lincRNAs), long intronic noncoding RNAs (intron-lncRNAs), and natural antisense transcripts (NATs) [21]. LncRNAs can affect gene expression by acting as cis- or trans-acting regulators [22, 23]. Li et al. identified more than 1,700 high-confidence lncRNAs among 20,163 putative lncRNAs in maize at a genome-wide level [24]. Huanca-Mamani et al. identified in a hyper-arid maize line 1,710 putative lncRNAs responsive to the combined stress of salt and boron, which showed an unusual higher expression relative to protein-coding genes under the stress conditions [25]. Lv et al. identified 1,077 differentially expressed lncRNAs in maize, including 509 transposable element (TE)-lncRNAs. The construction of co-expression networks further revealed 39 lncRNAs as major hubs that respond to abiotic stress, among which 18 were derived from TEs [26]. However, the reports on salt-responsive lncRNAs are still being discovered in maize.

By using two maize inbred lines with contrasting salt tolerance, we constructed 28 total RNA libraries across four stages of salt treatment for whole transcriptome sequencing. To accurately identify and characterize lncRNAs and their targets responding to salt stress, we developed a lncRNA and novel mRNA identification pipeline named NLncCirSmk by integrating different current methods. NLncCirSmk is based on the snakemake workflow management system, an open-source tool for creating reproducible and scalable data analyses [27]. NLncCirSmk is freely available on GitHub (https://github.com/Alipe2021/NLncCirSmk). By employing NLncCirSmk, the researchers can simultaneously identify differentially expressed lncRNAs, circRNAs and novel mRNAs from the whole transcriptome sequencing data.

## Results

### High-throughput sequencing and analysis workflow developing

**Fig. 1:**
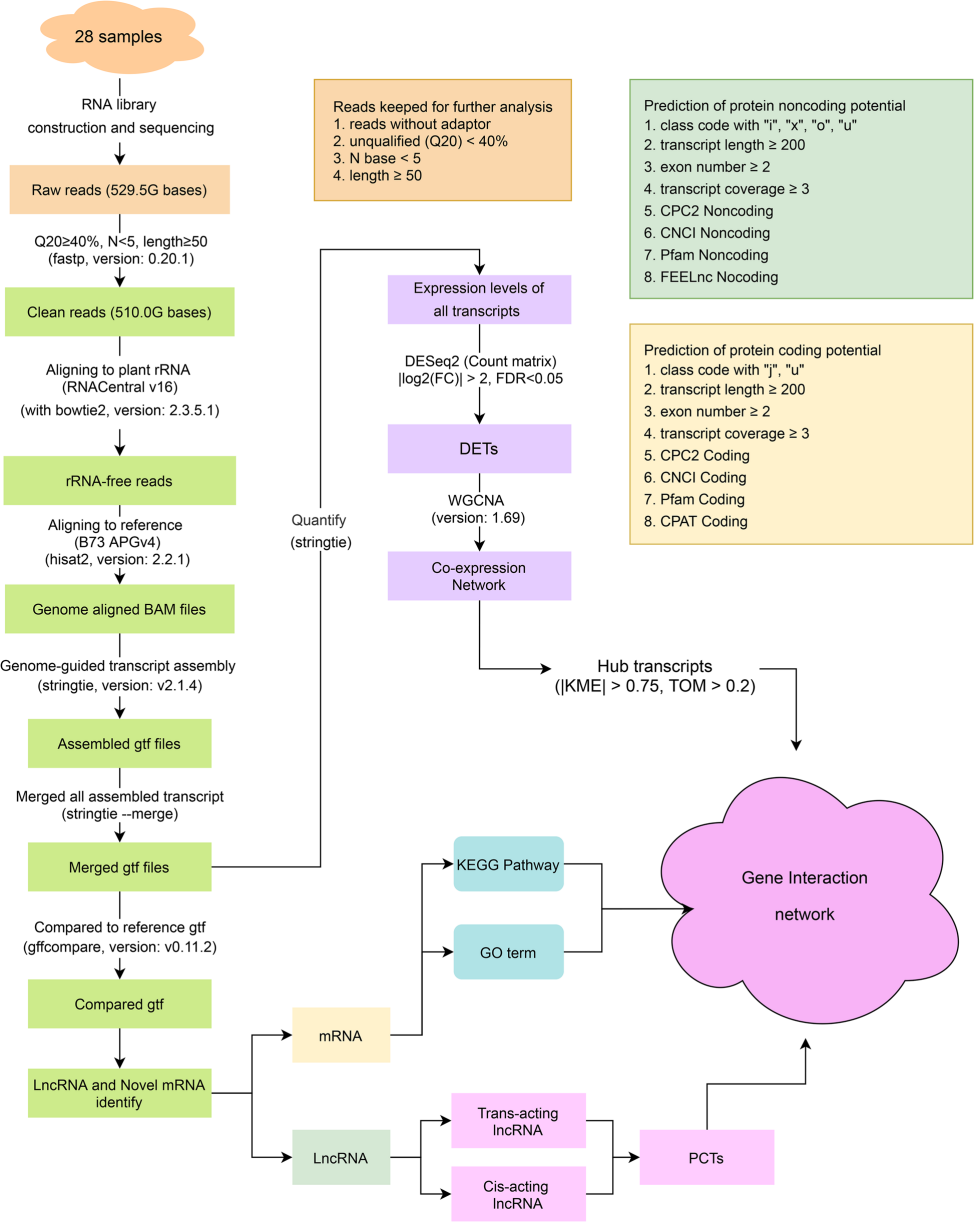
Workflow of bioinformatic analysis in our research. The figure shows the process of bioinformatic analysis in this study. The orange box illustrates the criteria for data quality control, the identification criteria for non-coding RNAs are listed in the pale green box, and the identification criteria for novel protein-coding RNAs are listed in the yellow box. The identification of lncRNAs and novel mRNAs are shown in the green rectangle. Differential expression analysis and gene co-expression analysis are shown in the purple rectangle. GO and KEGG analysis for mRNAs are displayed in the two cyan rounded rectangles. The gene regulation network shown in an irregular graph was constructed by integrating the results of differential expression analysis, co-expression analysis, and target prediction of lncRNAs

The lines L2010-3 and BML1234 were selected from an association panel of 330 maize inbred lines through a salt stress-tolerance test. Under salt stress, the salt-sensitive line BML1234 showed a more serious growth inhibition relative to the salt-tolerant line L2010-3, including decreased plant height and reduced biomass [28]. Under salt treatment, the two lines with contrasting salt tolerance were subjected to the construction of whole transcriptome libraries. Specifically, a total of 28 samples were collected from the tolerant line (L2010-3) and the sensitive line (BML1234) under CK (0, 6, 18, and 36 h) and salt treatment (6, 18, and 36 h), respectively, with two biological repetitions. To improve the efficiency of bioinformatic analysis, we developed a flexible, user-friendly, and modular RNA analysis workflow, named Novel mRNA, LncRNA, and CircRNA Analysis Snakemake Workflow (NLncCirSmk). It is based on the package management software Conda and the workflow management system Snakemake. The present workflow includes a complete pipeline for novel mRNA, lncRNA, and circRNA (developing) analysis (Fig. **1**). NLncCirSmk starts with the quality control of raw FASTQ files from paired sequencing data, going through optional trimming and rRNA filtering, alignment and assembly, identification of lncRNAs and novel mRNAs, and expression analysis. The workflow supports parallel computing and can greatly improve the speed of data processing. The source code of NLncCirSmk is available on GitHub (https://github.com/Alipe2021/NLncCirSmk).

### QC, rRNA filtering, transcripts assembly, and expression analysis

By performing high-throughput RNA-Seq, a total of 529.5G raw data were generated from the 28 whole transcriptome libraries. After removing low-quality reads, approximate 510.0 G clean bases were obtained. Among them, 93.01% reads had quality scores at the Q30 level (ratio of error rate ≤ 0.1%). By mapping the clean reads to the rRNA database, 1.89% to 9.70% clean reads were identified as rRNAs and were then filtered out (Table S1,Fig. **1**). The remaining rRNA-free reads were aligned to the maize reference genome (B73 RefGen_v4) [29], with the alignment ratio ranging from 33.56% to 66.33%. After transcriptome assembly, an integrated transcript set including 206,712 transcripts was reconstructed from all the 28 RNA-seq datasets. The sequencing and mapping statistics were summarized in Table S1.

**Fig. 2:**
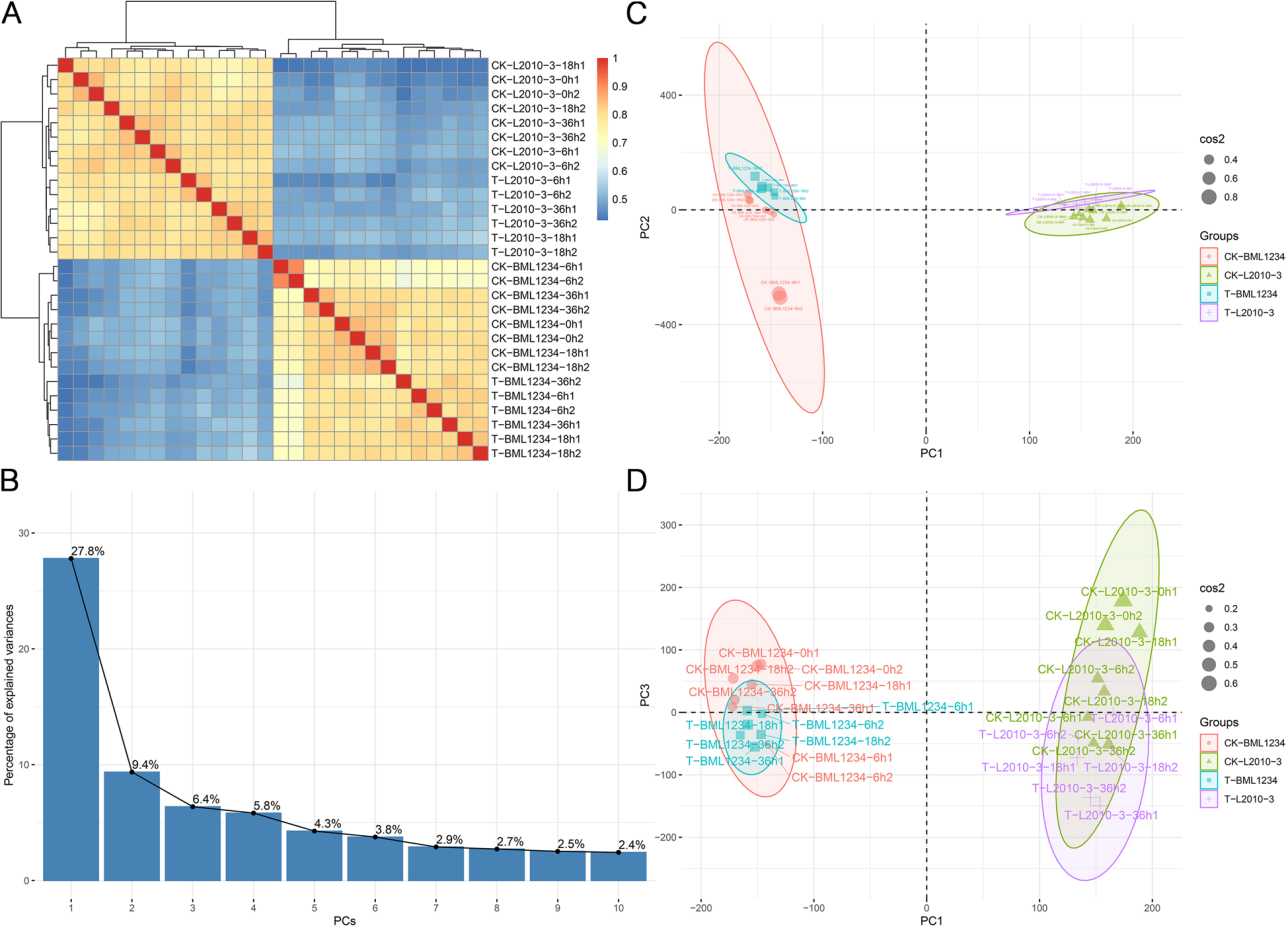
Relationships between transcriptome samples.** A** Correlation matrix heat map of transcript expression across all samples. Cluster dendrogram and spearman correlation coefficient heatmap are based on normalized TPM (transcripts per million mapped reads) values of expressed transcripts. The spearman correlation coefficients between different biological repetitions were calculated by the cor function in R software. Red indicates higher correlation; blue indicates lower correlation. The legend is added in the top right corner. **B** Scree plot of PCA. The first three principal components can explain more than 43% of the variability among the samples. **C**,** D** Principal component analysis (PCA) for the 28 samples. BML1234 in light the red circle. L2010-3 in the light blue triangle. The explained variances are shown in brackets. The cos2 of variables on all the dimensions are shown in different shape size. A high cos2 indicates a good representation of the variable on the principal component. PCA was performed using the R function “prcomp” based on the normalized read counts. The correlation heatmap and PCA diagram were drawn by the pheatmap package and factoextra package in R software, respectively

Correlation analysis showed that different biological repetitions had a high consistency and were clustered together (Fig. **2**A). PCA displayed that more than 43% of the variability in gene expression abundance among the samples were explained by the first three principal components (PCs) (Fig. **2**B). The overall gene expression levels of the 28 samples were evidently clustered into four different groups by different materials and treatment conditions. Especially, the obvious difference was observed between two contrasting lines. The samples from the salt-sensitive line BML1234 fell in the negative direction, whereas the samples from the salt-tolerant line L2010-3 resided in the positive direction of the PC1 axis. In the PC2 axis, the samples under salt treatment fell in the positive direction of the axis, and majority of the samples under normal conditions fell in the negative direction of the axis (Fig. **2**C). Moreover, the samples under normal conditions were mainly situated in the positive direction of the PC3 axis for both lines (Fig. **2**D). It revealed a remarkable difference in gene expression under the salt stress and normal condition.

### Identification and characterization of lncRNAs and novel mRNAs

**Fig. 3:**
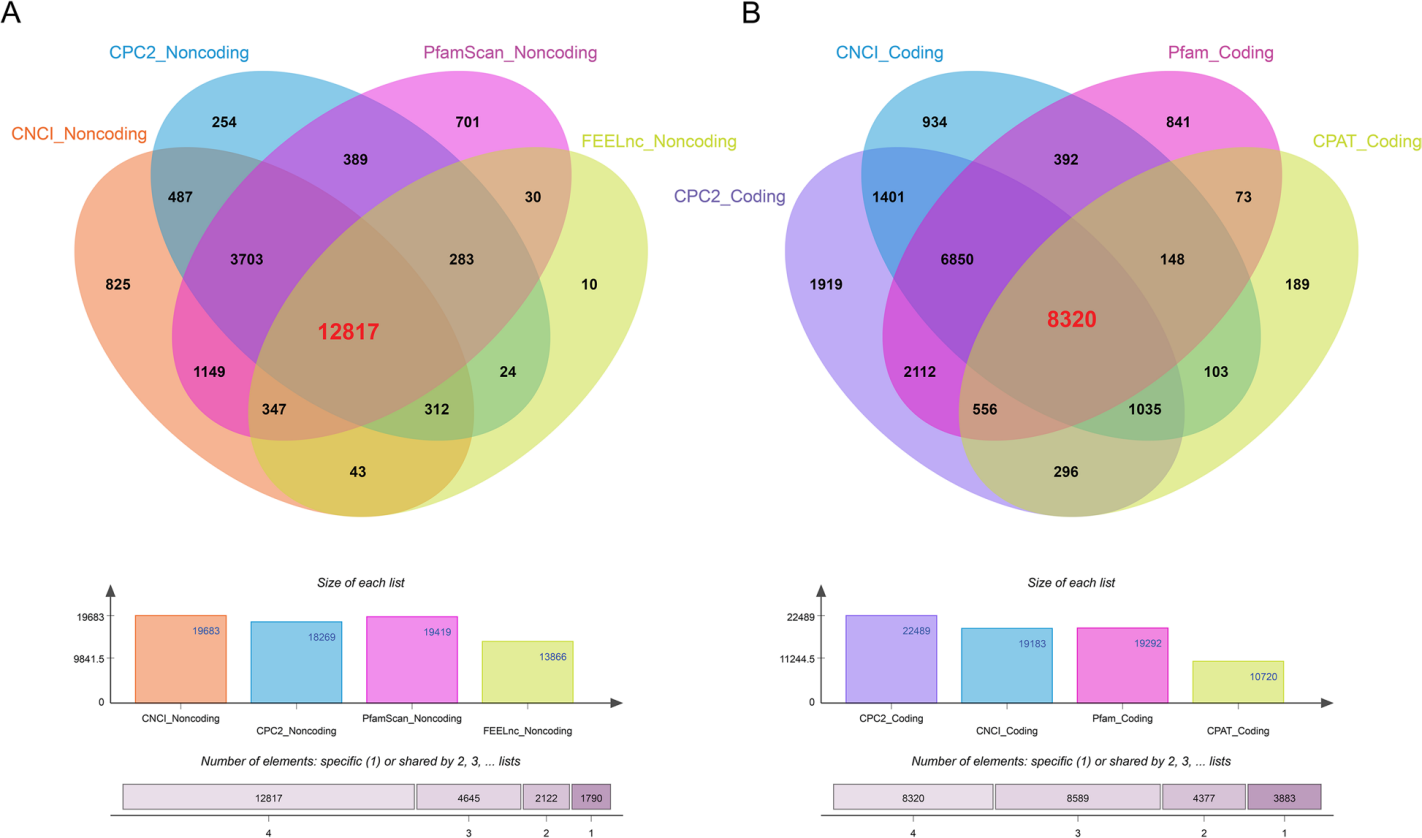
Prediction of protein-coding mRNA with different programs:** A** The top Venn diagram in the figure represents the prediction of protein-coding ability by different methods, and the bold red number represents the counts of the intersections of lncRNAs identified by Pfam, CNCI, CPC2, and FEElnc. The middle bar plot shows the counts of lncRNAs identified by each software. The bottom bin plot shows the number of elements in different combinations. **B** The top Venn diagram shows the prediction of protein-coding ability by four methods, and the bold red number represents the number of intersections of PCTs identified by four methods (CNCI, CPC2, FEELnc, and Pfam). The middle bar plot shows the counts of novel mRNA identified by each software. The bottom bin plot shows the number of elements in different combinations. The diagram was drawn by an online tools E Venn (http://www.ehbio.com/test/venn/#/)

**Fig. 4:**
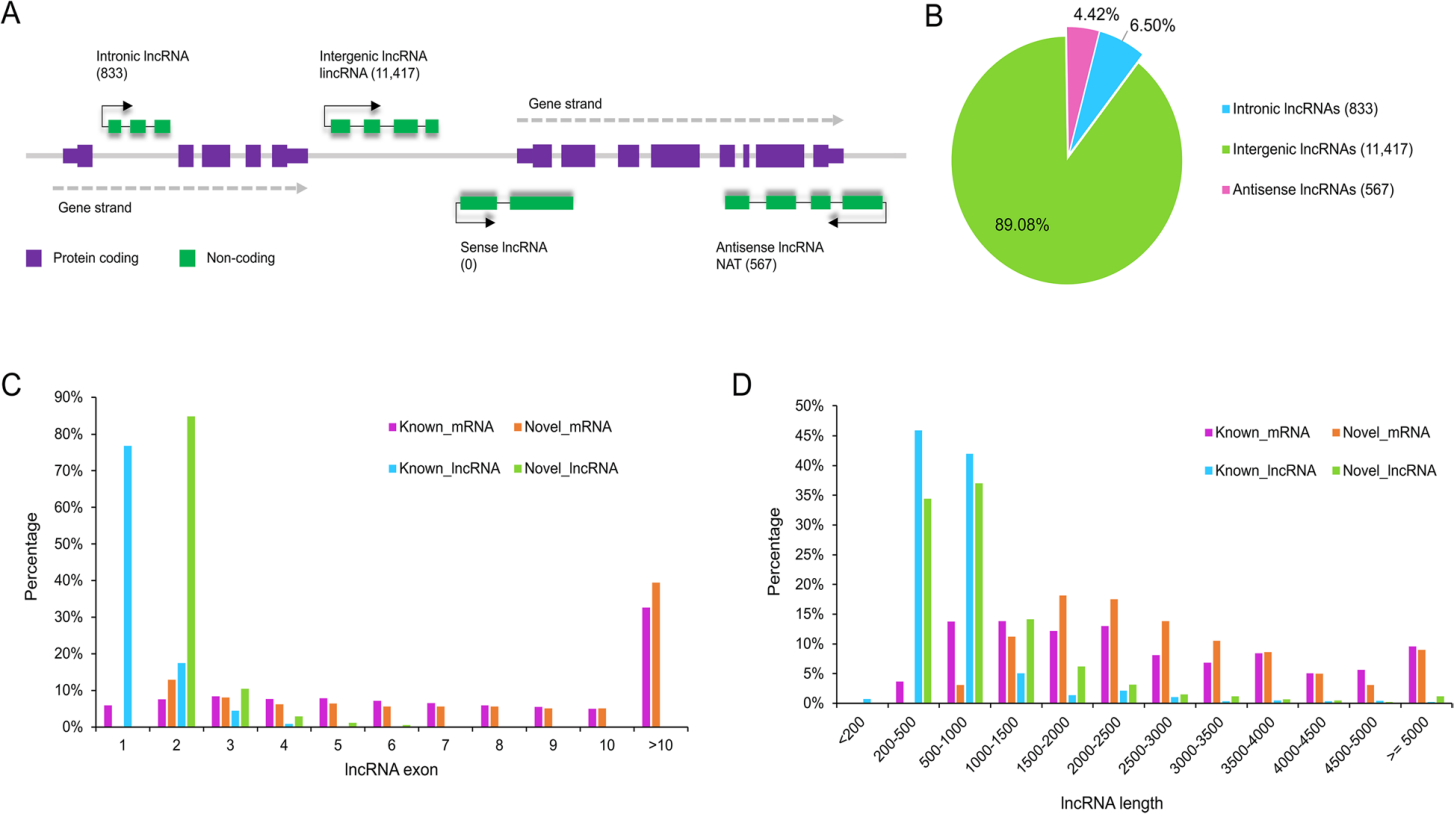
Statistics of the identified lncRNAs. **A**: Different types of lncRNAs based on their positional relationships to the adjacent genes. Green for lncRNAs and purple for protein-coding RNAs. The counts of different kinds of lncRNAs are shown in parentheses. The arrow direction indicates the transcription direction. **B** Bar plot shows the counts of different types of predicted lncRNAs. **C** Comparison of exon number percentages between the lncRNAs and mRNAs. Green for the predicted lncRNAs, blue for the known lncRNAs, purple for the known mRNAs, and orange for the novel predicted mRNAs in this study. **D** Comparison of transcript length between the lncRNAs and mRNAs

After filtering out the unqualified transcripts (length < 200, exon numbers < 2, and coverage < 3), 1,501, 696, 20,993, and 1,263 transcripts with class_code of "i", "o", "u", and "x" were detected, respectively, by comparing their genomic locations and directions to the reference transcripts. By removing potential protein coding transcripts (PCTs), a total of 12,817 common transcripts were defined as lncRNAs, including 422 known lncRNAs and 12,395 novel lncRNAs (Fig. **3**A). Among them, 833 (6.50%), 11,417 (89.08%), and 567 (4.42%) belonged to intronic-lncRNAs, lincRNAs, and antisense-lncRNAs, respectively (Fig. **4**A,4B). In addition, 8,320 transcripts were identified as novel PCTs (Fig. **3**B). According to the homologous annotations, 997 (12.39%) novel PCTs were involved in signal transduction, whereas 698 (8.67%) novel PCTs were related to posttranslational modification, protein turnover, and chaperones. The majority (1,867) of these novel PCTs were unknown transcripts (TableS2,FigureS1). In comparison with PCTs, 12,201 (95.19%) of the novel lncRNAs had fewer (< 3) exons and 11,755 (91.71%) had shorter (< 2000 bp) tags (Fig. **3**C,3D). The above findings were consistent with the previous studies in maize [24], *Cleistogenes songorica* [30], *Carya cathayensis* [31], and duckweed [32]. The detailed flowchart for identifying lncRNAs and novel PCTs was shown in Fig. **1**.

### Validation of the assembled transcripts by qRT-PCR

To validate the expression levels of the assembled transcripts from RNA-seq, six transcripts (*MSTRG.26461.5*, *MSTRG.28025.2*, *MSTRG.54905.9*, *MSTRG.61639.4*, *Zm00001d001353_T001* and *Zm00001d021924_T001*) were randomly subjected to expression examination by qRT-PCR. The Pearson’s correlation coefficient between RNA-Seq and qRT-PCR was calculated. The expression levels of these transcripts from RNA-seq data were significantly correlated with those using qRT-PCR (*R*^2^ ≥ 0.6112, *P* < 0.001) (**Table S3**, **Figure S2**), indicating that the expression profile based on RNA-seq data was reliable in the present study.

### LncRNAs and PCTs responding to salt stress

**Fig. 5:**
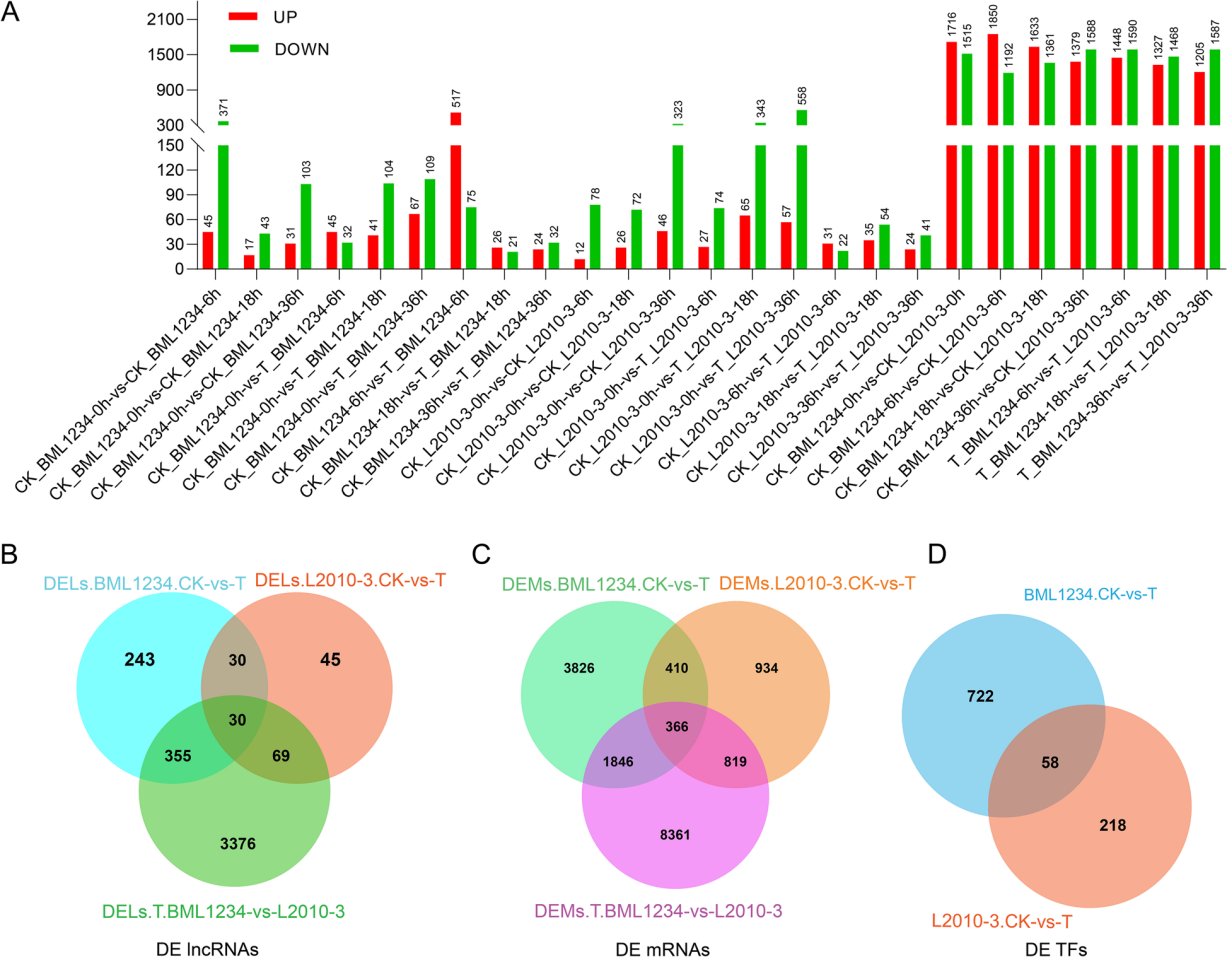
**Distributions of differentially expressed lncRNAs. A)** Bar graph of up- and down-regulated lncRNAs from pairwise comparison. **B)** Venn diagram shows the numbers of DELs in different comparison groups. Blue represents the total DETs in BML234 between normal and salt treatment conditions. Brown indicates the total DELs between normal and salt treatment conditions in L2010-3. Green represents the total DELs under salt stress between different lines. **C)** Venn diagram shows the numbers of DEMs in different comparison groups. **D)** Venn diagram shows the numbers of DE-TFs in different comparison groups

The differentially expressed lncRNAs (DELs) were identified according to the criteria: |log2FC|> 2, *P*-adjust < 0.01. In the salt-sensitive line BML1234, 592 (517 upregulated and 75 downregulated), 47 (26 upregulated and 21 downregulated), and 56 (24 upregulated and 32 downregulated) DELs were detected at 6, 18, and 36 h under salt stress, respectively (Fig. **5**A,Fig. S3A-C). In the salt-tolerance line L2010-3, 53 (31 upregulated and 22 downregulated), 89 (35 upregulated and 54 downregulated), and 65 (24 upregulated and 41 downregulated) DELs were detected at each salt treatment stage (Fig. **5**A,Fig. S3D-F). Among these DELs, 598 and 114 were specifically responsive to salt stress in BML1234 and L2010-3, respectively, whereas 30 DELs were common between the two lines (Fig. **5**B). The DELs in BML1234 between normal and salt stress conditions at 6 h was much more than those in the other samples, implicating that the response of lncRNAs to salt stress mainly occurred at the early stage of the salt treatment in the sensitive line. Besides, the expression of lncRNAs in the sensitive material was more easily affected by the salt stress compared with the tolerant line. By comparing the lncRNA expression levels at a given treatment stage between the two lines, we identified 3,038, 2,795, and 2,792 DELs at 6, 18, and 36 h of salt treatment (Fig. **5**A,Fig. S3G-I). Similarly, a total of 20,107 differentially expressed transcripts (DETs) (13,911 known mRNAs, 4,511 lncRNAs, and 1,685 novel mRNAs) were found between the two lines under normal conditions. These suggested that the contrasting genetic backgrounds of the two lines generated considerable variations in transcript expression abundance.

In addition, we obtained a total of 7,835 differentially expressed mRNAs (DEMs), which presented different salt-stress responses between the two lines, involving 5,672 and 1,753 specific DEMs in BML1234 and L2010-3, respectively. Among the 7,835 DEMs, 821 were newly identified PCTs, involving 507 and 272 specific novel PCTs in BML1234 and L2010-3, respectively (Fig. **5**C). Furthermore, 998 DEMs were defined as TFs, among which *C2H2*, *WD40*, *MYB*, *PHD*, and *bZIP* families were the top five largest TF families, individually including 151, 126, 81, 76, and 38 DEMs (Table S4). At each comparison stage between CK and treatment conditions, more differentially expressed TFs (DE-TFs) were detected in BML1234 than in L2010-3 (Fig. **5**D).

### Predicted lncRNA targets responding to salt stress

To further address the roles of the 742 potential salt stress-responsive DELs, we identified from all the DEMs the potential cis- and trans- target transcripts. In results, 41 DEMs were located between the 100 kb upstream and downstream of the 742 DELs and were significantly correlated (Pearson correlation coefficient, |PCC|> 0.6, *P*-value < 0.05) with their corresponding lncRNAs, which were thereby defined as the cis-regulated target transcripts (Table S5). These 41 cis-transcripts were regulated by 35 regulatory DELs. Meanwhile, 81 DEMs including 5 novel mRNAs with the free energy < -0.2, |PCC|> 0.8, and *P*-value < 0.01 were identified as the trans-target transcripts of 58 DELs (**Table S6**). In total, 168 lncRNA-mRNA pairs including 123 trans- and 45 cis- pairs were detected, which were speculated to be involved in salt tolerance in maize seedlings. These target transcripts were categorized into 17 COGs categories and the top 5 categories were annotated as “function unknown”, “transcription”, “intracellular trafficking, secretion, and vesicular transport”, “inorganic ion transport and metabolism”, and “translation, ribosomal structure and biogenesis”. In addition, 11 transcripts belonging to 7 TF families were detected, including *C2H2* (*Zm00001d016139_T001* and *Zm00001d049767_T001*), *HAS (Zm00001d044281_T003* and *Zm00001d044283_T005*), *Hap3/NF-YB* (*Zm00001d032328_T005*), *MYB-HB-like* (*Zm00001d011691_T002*, *Zm00001d044281_T003*, and *Zm00001d044283_T005*), *WD40-like* (*Zm00001d011920_T002*, *Zm00001d040038_T003*, and *Zm00001d046587_T028*), *WRKY* (*Zm00001d007329_T001*), and *bHLH* families (*Zm00001d043706_T001*). These TFs were previously reported to respond to salt stress, growth and development in plants [33–37]. KEGG enrichment analysis indicated that “RNA polymerase”, “oxidative phosphorylation”, and “protein export” pathways were significantly enriched with the target transcripts (Table S7). GO analysis uncovered 11 (integral component of plasma membrane, DNA-directed RNA polymerase III complex, and others), 42 (calcium-transporting ATPase activity, cation-transporting ATPase activity, and others), and 14 (single fertilization, ATP hydrolysis coupled transmembrane transport, and others) terms as the most significantly enriched GO terms in cellular component (CC), molecular function (MF), and biological process (BP), respectively (Table S8).

### Salinity stress-responsive modules in WGCNA

**Fig. 6:**
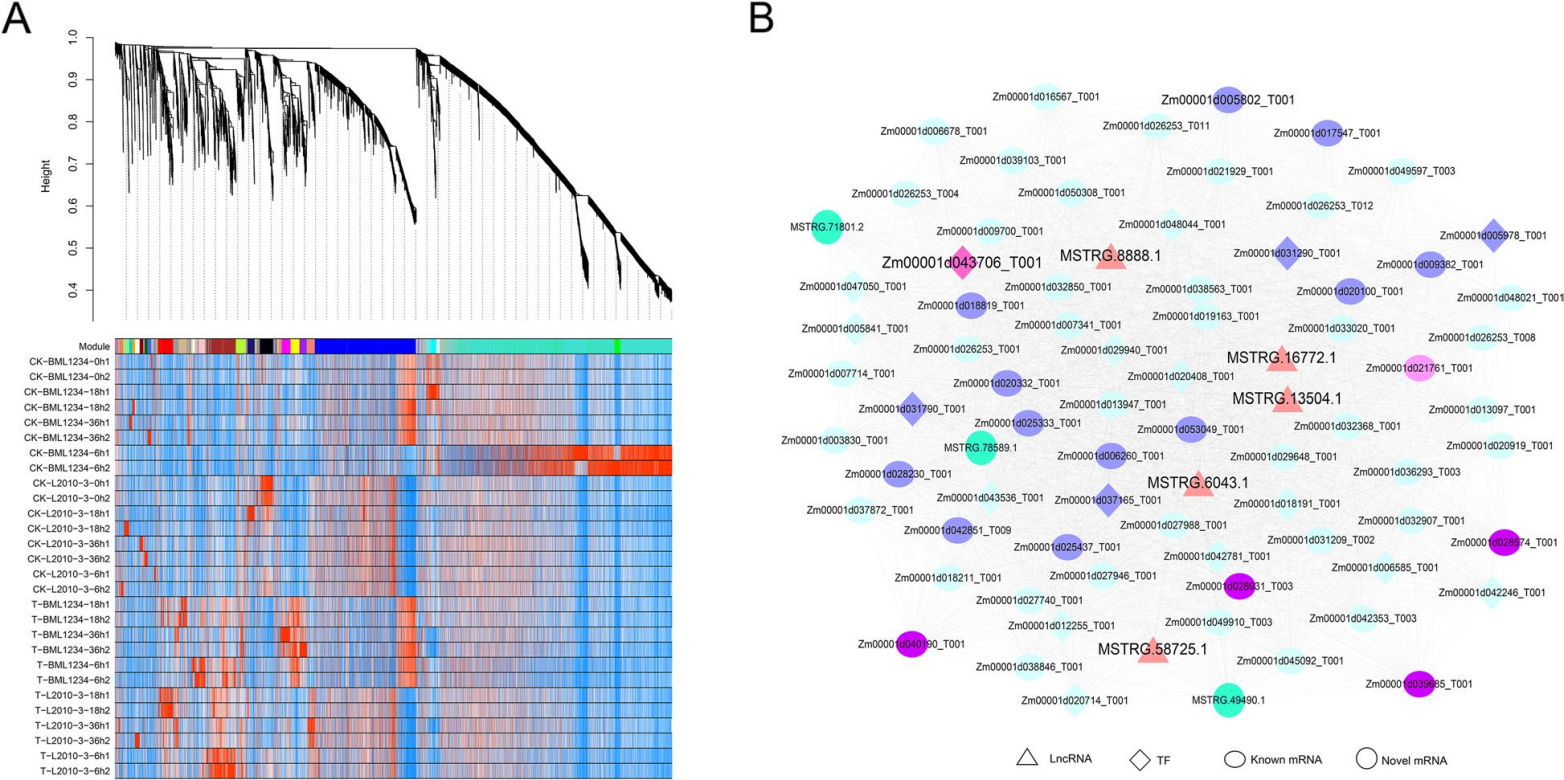
**Co-expression modules of DETs and network dynamics in response to salt stress in maize seedling. A**) Transcripts hierarchical clustering tree of different modules. Each major tree branch stands for one module, each leaf in the tree represents one transcript, different modules are labelled with different colors. **B)** Detailed network dynamics of nodes in brown module. Dark blue ellipses represent the top five connected mRNAs for each lncRNA, pink ellipse displays one of top five connected mRNAs to the *bHLH*, dark purple ellipses stand for common mRNAs in top five connected mRNAs of *bHLH* and lncRNAs, light blue shows other mRNAs

Co-expression modules have been used to exhibit the interaction relationships between different function-associated genes [38, 39]. In total, 8,577 DETs including 7,835 DE-PCTs and 742 DELs were used for constructing the co-expression modules via WGCNA (Table S9). The soft-threshold power of β was determined as 4 (Fig. S4) when the scale-free topology index was 0.95. In total, 28 distinct modules were built with the parameters (deepSplit = 2 and minModuleSize = 30), which were labelled with different colors (Fig. **6**A). The number of DETs in each module ranged from 37 to 3,237 and 6,748 (83.28%) DETs were classified into the top ten modules. Moreover, 621 (86.73%) DELs were clustered into the blue, turquoise, black, yellow, red, and brown modules. To identify the biological function of the DELs in each co-expressed module, we executed KEGG pathway and GO enrichment analyses. The transcripts in the turquoise, red, and brown modules were significantly (FDR < 0.05) enriched in 3 (“ribosome”, “proteasome”, and “ribosome biogenesis in eukaryotes”), 4 (“fatty acid elongation”, “cutin, suberine and wax biosynthesis”, “biosynthesis of secondary metabolites”, and “linoleic acid metabolism”), and 10 (“metabolic pathways”, “plant hormone signal transduction”, and others) pathways, respectively. GO analysis showed that 128 ribosome-relevant terms, 88 abiotic stimulus response-related terms, 72 transferase activity-associated terms, and 4 glyoxylate cycle-relevant terms were significantly (FDR < 0.05) enriched in the turquoise, red, brown, and yellow modules, respectively. Previous studies reported that plant response to salt stress involves abiotic stimulus response and transferase participation [19, 40]. Therefore, we further focused on the red and brown modules to identify the hub transcripts. The KME of the transcripts in these modules were calculated by the signedKME function in R package. In total, 5 lncRNAs (*MSTRG.13504.1, MSTRG.16772.1, MSTRG.58725.1, MSTRG.6043.1, and MSTRG.8888.1*) and 231 PCTs were identified as the hub transcripts (|KME|> 0.75, TOM > 0.2) in brown module. Interestingly, 14 PCTs were the target transcripts of 5 DELs in the hub transcript set, including the *bHLH* TF (*Zm00001d043706_T001*)/ *MSTRG.8888.1* pair. Meanwhile, 85 hub transcripts including 2 lncRNAs (*MSTRG.62146.4* and *MSTRG.68516.1*) and 83 mRNAs were detected in the red module, of which 25 transcripts were significantly enriched in the results of GO analysis.

### Regulatory network mediated by lncRNAs and their target TFs

Since some TFs were identified as the targets of lncRNAs in the brown module, we further constructed the regulatory networks of salinity response mediated by lncRNAs and their target TFs. In the brown module, a network with 79 nodes and 3,082 edges was established, which contained 5 DELs, 18 TFs, 3 novel mRNAs, and 53 known DEMs. Notably, the *bHLH* TF *Zm00001d043706_T001* was identified as a hub (KME = 0.89, TOM = 0.22) gene in the module, which was trans-regulated by the lncRNA *MSTRG.8888.1*. Meanwhile, *Zm00001d043706_T001* have significant co-expression relationships with 73 PCTs, of which 19 contained 1–8 bHLH binding motifs (Table S10). The top five co-expressed mRNAs of *Zm00001d043706_T001* included *Zm00001d021761_T001* (*MYB-transcription factor 105*, *MYB105*), *Zm00001d028574_T001* (*protein-serine/threonine phosphatase 14*, *PP2C14*), *Zm00001d028931_T003* (*galactinol synthase 1*, *GOLS1*), *Zm00001d039685_T001* (*raffinose synthase 1*, *RAFS1*), and *Zm00001d040190_T001* (*hydroxyproline-rich glycoprotein*, *HRGP*) (Fig. **6**B). These genes have been previously reported to correlate with the response of salt or other abiotic stress [41–44].

## Discussion

In this study, two maize inbred lines (BML1234 and L2010-3) with contrasting salt tolerance were used for exploring the lncRNAs and their target transcripts involved in salt stress response. Our previous studies indicated that the two lines with different backgrounds showed different phenotypes under salt treatment of 150 mM NaCl concentration [28, 45]. Generally, the shoot and root growth was more seriously inhibited under salt treatment in the salt-sensitive line BML1234 compared with the salt-tolerant line L2010-3 [28]. A total of 178 K single nucleotide polymorphisms (SNPs) existed between the two lines [39]. In the present study, considerable variations in transcript expression levels were found between BML1234 and L2010-3, which coincided with the large genetic variations between the two lines. Salt stress was one of main abiotic stresses, which caused a major problem for plant growth and production. More and more evidence supported that lncRNAs play significant roles in stress response [41, 46–48]. Although some functional genes such as *ZmNHX*, *ZmHTK*, *MIP*, and *SnRK2* have been proven to participate in the response to salt stress in plants [6, 7, 49, 50], only a few lncRNAs have been reported to involve salinity stress at a whole transcriptome level. To reveal the salt responsive lncRNAs and the mechanism underlying salt tolerance in maize, we first identified lncRNAs from maize seedling root at different salt treatment stages and normal conditions using whole transcriptome sequencing. Through a strict bioinformatic pipeline, we uncovered a total of 12,718 high-confidence lncRNAs. Compared with protein-coding genes, lncRNAs were shorter in length and had fewer exons in structures, which were consistent with the previous reports [51, 52]. Then, we executed the comparative transcriptomic analysis, which identified more than 700 differential DELs between two lines with distinct salt tolerance. Most of the salt-responsive lncRNAs showed the differential expressions at the early stages of salt stress, especially in salt-sensitive line BML1234, which partially explained for the phenotypes of a more serious growth inhibition in BML1234.

The expression of functional genes was regulated by various factors, such as TFs, miRNAs, and lncRNAs [53–55]. As transcriptional regulators, lncRNAs affect the expression of functional genes directly or indirectly [56]. In the present study, we identified 45 cis- and 123 trans- lncRNA-mRNA pairs. Most of the lncRNA-mRNA pairs showed positive correlations in expression levels, whereas only 14 pairs (10 cis- and 4 trans-) had negative correlations. To further recognize the function of these DELs under salt stress, we performed KEGG pathway and GO term enrichment analysis for the target transcripts of the DELs. Some salt stress-responsive pathways and GO terms such as “oxidative phosphorylation” pathway and “calcium-transporting ATPase activity” term [57, 58] were significantly enriched with the target transcripts. These findings suggested that the DELs were involved in salt response of maize seedlings and contributed to the difference of salt tolerance between these two lines.

**Fig. 7:**
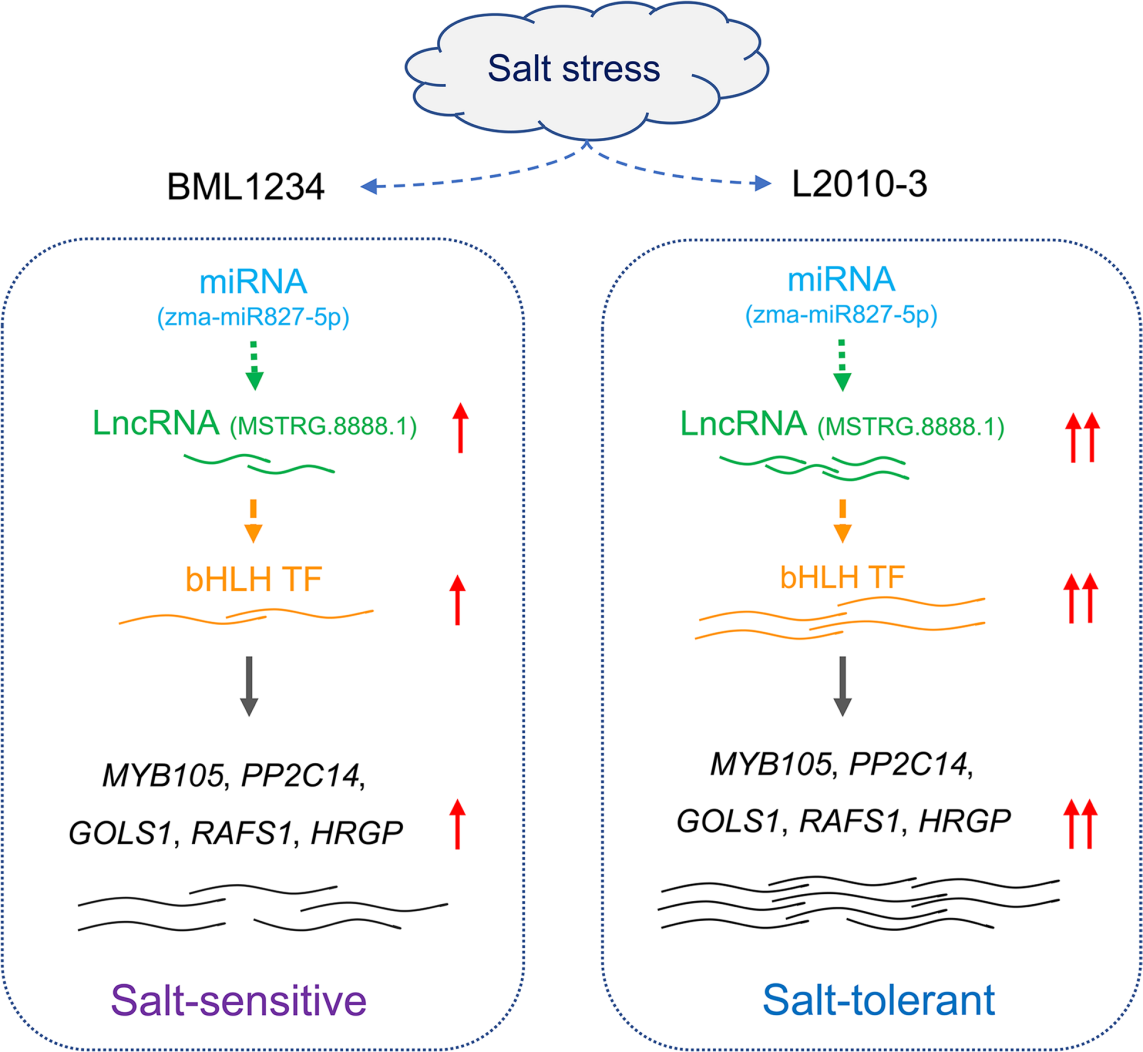
**A predicted module for MSTRG.8888.1 mediated salt resistance.** At the early stage of salt stress, *zma-miR827-5p* responded to the salt signal and modulated the *MSTRG.8888.1* at the post-transcription level; then the modified transcript abundance of *MSTRG.8888.1* affected the transcription and translation of the *bHLH*; the altered bHLH protein level induced the upregulated expression of five salt tolerance-related functional transcripts (*MYB105*, *PPR2C14*, *GOLS1*, *RAFS1*, and *HRGP*) by binding their promoters. The distinct responses of *MSTRG.8888.1* to salt stress may partly account for the difference in salt tolerance between the two lines. The number of wavy lines showed the relative expression levels (RELs) of transcripts (the transcript ratios between salt stress and normal conditions). The red arrows represent the upregulated expression of corresponding genes under salt stress. The double arrows showed a higher REL in comparison to the single arrow

The previous studies showed that the interaction relationships between lncRNAs and TFs may ameliorate the expression levels of their target functional genes [59]. Therefore, we built a co-expression network to further investigate the relationships among the lncRNAs, TFs, and other mRNAs. In the lncRNA-TFs-mRNA interaction networks, we found a total of 18 TFs were co-expressed with 5 lncRNAs and 56 mRNAs. Among the 5 lncRNAs, a hub lncRNA *MSTRG.8888.1* acted as a trans-regulator and regulated the expression of another hub transcript *bHLH* TF *Zm00001d043706_T001*. The *bHLH* family was one of the largest families of transcription factors in plants [60], involved in plant response to diverse abiotic stresses, such as heavy metal toxicity, osmotic damages, drought, chilling, and salinity [61–63]. In this study, the top five functional mRNA co-expressed with *Zm00001d043706_T001* contained *Zm00001d021761_T001*, *Zm00001d028574_T001*, *Zm00001d028931_T003*, *Zm00001d039685_T001*, and *Zm00001d040190_T001. Zm00001d028931_T003* that encodes a galactinol synthase, which has been extensively reported to confer salt tolerance in plants by mediating the biosynthesis of galactinol and raffinose family oligosaccharides [41, 64]. Consistently, our present study found that the *Zm00001d028931_T003* was significantly up-regulated under salt treatment with a higher expression level in the salt-tolerance line. Remarkably, the promoter of *Zm00001d028931_T003* contained two G-box (CACG[TA]C) motifs, which are the typical *bHLH* TF-binding motifs. This provided the evidence that *Zm00001d028931_T003* was regulated by the *bHLH* TF *Zm00001d043706_T001*. Collectively, we constructed the regulatory network of salt-stress response, which was mediated by *MSTRG.8888.1/ Zm00001d043706_T001*. Some lncRNAs have been previously reported to act as miRNA targets or decoys, involving the regulation of gene expression [65]. Using a plant microRNA endogenous target mimics prediction tool, psMimic [66], five of these 742 DELs were identified as potential miRNA decoys and bound by six miRNAs, forming 12 miRNA-lncRNA duplexes (Table S11). In these pairs, each of the three DELs *MSTRG.15598.1*, *MSTRG.15598.3*, and *MSTRG.7211.8* adsorbed the miRNAs *zma-miR399b-5p*, *zma-miR399d-5p*, and *zma-miR399i-5p*. Meanwhile, the DELs *MSTRG.57825.1* and *MSTRG.57690.7* acted as the sponges of one (*zma-miR160d-3p*) and two (*zma-miR167h-3p* and *zma-miR167i-3p*) miRNAs, respectively. Besides, using the miRbase (version 22.1) [67] and psRNATarget web server [68], we predicted the possible miRNA targets from the 742 DELs. In total, 322 lncRNAs were identified as the potential targets of 301 mature miRNAs (Table S12). Among them, the lncRNA *MSTRG.8888.1* was distinguished as a possible target of *zma-miR827-5p*. Notably, *miR827* has been extensively reported to regulate salt tolerance in plant species including cotton [69], banana [70], and *Arabidopsis thaliana* [71]. Based on these evidences, we present a model to summarize the putative regulatory pathway mediated by *MSTRG.8888.1* (Fig. **7**). As an upstream effector of this pathway, *zma-miR827-5p* responded to the signal of salt stress and regulated the *MSTRG.8888.1* at the post-transcription level; then the changed *MSTRG.8888.1* expression affected the transcription and translation of the *bHLH*; the altered protein abundance of the bHLH subsequently induced the upregulated expression of the five salt tolerance-related functional genes by binding their promoters (Fig. **7**). Moreover, the differential responses of *MSTRG.8888.1* to salt stress may partly account for the disparity in salt tolerance between the two lines (Fig. **7**). The Snakemake workflow management system is a tool to create reproducible and scalable data analysis pipelines. Based on the Snakemake, we developed the NLncCirSmk to build an efficient, flexible, and reproducible bioinformatic analysis pipeline. The NLncCirSmk could deal with numbers of samples at the same time with a modifiable profile. To reduce the false positives of lncRNAs identification, different approaches had been integrated into NLncCirSmk.

Collectively, our results provide new sights into further revelation of lncRNA function in maize tolerance to salt stress.

## Conclusions

In conclusion, we identified 12,817 lncRNAs and 8,320 novel mRNAs in two maize lines with contrasting salt tolerance by using our developed bioinformatic pipeline. In total, 742 DELs were identified as the salt-tolerance transcripts. Among the five hub lncRNAs, *MSTRG.8888.1* acted as a trans-regulatory and affected the expression of salt tolerance-relevant genes by targeting the *bHLH* transcript, *Zm00001d043706_T001*. Meanwhile, *MSTRG.8888.1* was a potential target of *miR827* that has been reported to involve the salt tolerance in other plant species. Based on these evidences, we present a model to summarize the putative regulatory pathway mediated by *MSTRG.8888.1*. Our results might expand our horizon for understanding the salt-tolerance mechanism regulated by lncRNAs in maize.

## Methods

### Plant materials and salt treatment

Two maize inbred lines, BML1234 (a salt-sensitive line) and L2010-3 (a salt-tolerant line), were used in this study, which have been described in our previous study [29]. For each line, the seeds were surface-sterilized using 10% (v/v) H_2_O_2_ for 15 min and then rinsed three times with distilled water. After that, the seeds were germinated on filter paper saturated with distilled water and then grown at 26 °C under 14-h day/10-h night conditions.

Uniform seedlings with two leaves were randomly divided into two groups: CK (cultivated in Hoagland’s solutions) and salt treatment (cultivated in Hoagland’s solutions with 150 mM NaCl). These seedlings were then cultured in a growth room with a relative humidity of 70% at 26℃, and a cycle of 14-h day/10-h night. At 0, 6, 18, and 36 h, the mixed roots from three seedlings of each line were individually collected as the samples for whole transcriptome sequencing, with two biological repetitions.

### RNA isolation, sequencing, and quality control

Referring to the manufacturer’s instructions, total RNA of each sample was extracted using the HiPure Plant RNA Maxi Kit (Magen Company, Guangzhou). The quality and purity of RNA were analyzed with a 2100 Bioanalyzer and RNA 6000 Nano kit 5067–1511 (Agilent, CA, United S). Ribosome RNA (rRNA) was filtered by Illumina Ribo-Zero rRNA Removal Kit. RNA libraries were constructed by the Illumina sequencing platform and sequenced on a Hiseq 4000 system (Illumina) using the PE150 method. All raw data were deposited in Genome Sequence Archive (GSA) in National Genomics Data Center (NGDC) database with the accession number CRA003872.

Raw reads were filtered with fastp [72] to remove the low-quality reads, polyN-containing reads and adapter reads. Using bowtie2 [73], the remaining high-quality reads were further aligned in the plant rRNA database (RNACentral v16) [74] to remove rRNA sequences.

### Genome alignment, isoform assembly and isoform expression calculation

After filtering rRNA, the remaining reads were aligned to the maize genome (B73 RefGen_v4) using hisat2 [75], allowing up to 2 mismatches. Then, we assembled the transcriptome using stringtie program with params ‘-m 200 -a 10 –conservative -g 50 -u’). Subsequently, the program “stringtie merge” (params: -m 200 -c 3) was used to merge the information of all transcripts, generating the integrated transcript information [76]. The expression level of each isoform was calculated by re-assembling with integrated transcript information. The read counts and transcripts per million (TPM) matrixes were directly extracted from the files generated by a Python script (prepDE.py, provided by stringtie program). After filtering the low-expression transcripts by a customized R script, the counts matrix was used to perform differential expression analysis and the TPM matrix was used to conduct correlation analysis, principal component analysis (PCA), and co-expression network construction.

### Identification of pseudo lncRNAs and novel protein-coding transcripts

To identify putative lncRNAs and novel PCTs, we used a rigorous set of criteria to annotate the assembled transcripts. First, a flexible extraction of long non-coding RNAs tool (FEELnc) was employed to detect potential lncRNAs based on a random forest model [77]. Then, the integrated transcripts were compared with the maize reference transcripts by the gffcompare program [78]. The following steps were performed to identify the lncRNAs from the transcripts based on their characteristics: (1) transcripts with class_code of "i", "u", “x” and "o" were selected; (2) transcripts with a length < 200 bp and an exon count < 2 were removed; (3) transcripts with a TPM ≥ 1 were selected; (4) transcripts that did not pass the protein-coding-score test were eliminated using the Coding Potential Calculator (CPC version2) [79] and Coding-Non-Coding Index (CNCI) [80]; (5) known mRNA and transcripts with protein-coding domain in Pfam databases were removed [81]. The intersections of non-coding transcripts identified by Pfam, CNCI, CPC and FEElnc were considered as the putative lncRNAs.

Additionally, we used the transcripts with class_code of “j” and “u” for the prediction of novel protein-coding transcripts. After predicting candidate coding regions within transcripts by TransDecoder software (https://github.com/TransDecoder), we calculated the coding potential score of each transcript using CPC2, CNCI, PfamScan, and an alignment-free method Coding Potential Assessment Tool (CPAT) [82]. Those transcripts that were simultaneously defined as coding mRNAs by four methods were recognized as novel mRNAs. The Clusters of Orthologous Genes (COG) categories and functional annotations were then predicted using an online tool eggnog-mapper (http://eggnog-mapper.embl.de/) [83].

### Correlation analysis, PCA, and differential expression analysis

To evaluate the relationship between samples, we performed the correlation analysis and PCA for the 28 samples. First, we filtered out the transcripts with lower expression levels, which may be caused by assembly errors. Then, the correlation analysis and PCA were carried out with the cor function using the Pearson method and prcomp packages in R, respectively. Finally, Deseq2 were used to detect differentially expressed lncRNAs and novel protein-coding transcripts. Transcripts with |log2FC|> 2 and FDR < 0.01 were identified as significant DETs [84]. A Perl script was used to fetch and count the DETs in the different comparison groups.

### Prediction of lncRNA targets

To determine the cis-target transcripts of lncRNAs, we searched for PCTs within 100 Kb upstream and 100 Kb downstream of the lncRNAs [53]. The Pearson correlation coefficient (PCC) between the lncRNA and the corresponding PCT was then calculated based on their expression levels. The PCTs that met the strict standards (|PCC|> 0.6, *P* < 0.05) were considered as cis-target transcripts of the lncRNAs.

Furthermore, we used the LncTar program to predict the trans-targets of lncRNAs based on complementary base pairing [85]. The transcript was considered a trans-target of the lncRNA, when the free energy of pairing sites between transcript and lncRNA was lower than the threshold of standardized free energy (ndG <  − 0.2) [51]. Besides, the PCC between the lncRNA and the corresponding transcript was calculated. Those mRNAs with |PCC|> 0.8 and *P*-value < 0.01 in lncRNA-mRNA pairs were defined as the putative trans-target mRNAs of the lncRNAs [86].

### Identification of transcription factors

TFs have been proved to play crucial roles in maize response to salt stress [87]. In the present study, an online tool PlantTFcat [88] was used to conduct TF analysis for all the DETs.

### Weighted gene co-expression network construction

The WGCNA was executed with the WGCNA (v1.69) package in R [89] based on the normalized expressions of DETs. The transcripts with the eigengene connectivity (KME) > 0.75 or < -0.75 and topological overlap measure (TOM) > 0.2 were defined as the hub transcripts.

### Quantitative real-time PCR

To verify the sequencing results, we randomly selected six transcripts for qRT-PCR. The primer pairs for qRT-PCR were designed using the Primer 5.0 software and are shown in Table S3. The qRT-PCR was carried out with an Applied Biosystems 7500 Real-Time PCR System with three biological repetitions. The reaction program was as follows: 2 min at 98 °C, 2 s at 98 °C, 10 s at 59 °C, 40 cycles. A thermal denaturing step was then performed for generation of the melting curves for amplification specificity verification. The maize *Actin1* gene (*Zm00001d010159*) was selected as the reference for normalizing the gene expression. The 2^−△△ct^ method was used for calculating the relative expression levels of target genes.

### KEGG Pathway and GO term enrichment analysis

Gene Ontology (GO) and Kyoto Encyclopedia of Genes and Genomes (KEGG) pathway enrichment analyses were performed using the OmicShare, a free online platform for data analysis (www.omicshare.com/tools).

### Construction of regulatory network

In the module enriched with the transcripts involving abiotic stress-response and transferase activity, we combined the hub lncRNAs, the corresponding target transcripts, and the other transcripts in the module and constructed the regulation network mediated by the hub lncRNAs. Cytoscape 3.7.1 [90] was then utilized to draw the putative interaction network.

### Statements

The two maize lines used in this study were provided by Sichuan Agricultural University and comply with relevant institutional, national, and international guidelines and legislation.

## Supplementary Information

**Additional file 1: Figure S1.** COGs annotation for novel mRNAs. Different alphabets indicate various COGs. Detailed information of COGs can be found in the COG database (https://www.ncbi.nlm.nih.gov/research/cog/). **Figure S2.** qRT-PCR validation of identified lncRNAs and PCTs. **Figure S3.** Venn diagrams for DELs in different comparable groups. **Figure S4.** Determination of soft thresholding power in the WGCNA. The left panel shows the influence of soft threshold power on the scale free topological fit index; the right panel shows the influence of soft threshold power on the average connectivity.

**Additional file 2: Table S1.** Sequencing and mapping statistics for each sample. **Table S2.** Annotations of novel mRNAs. **Table S3.** Primer sequences for qRT-PCR. **Table S4.** Predicted TFs in the DEMs. **Table S5.** Cis-target transcripts of DELs. **Table S6.** Trans-target transcripts of DELs. **Table S7.** KEGG enrichment analysis for target mRNAs. **Table S8.** GO enrichment analysis for target mRNAs. **Table S9.** Expressions of DETs for WGCNA. **Table S10.** The 19 co-expressed genes of the bHLH TF, which contained bHLH binding motifs. **Table S11.** Prediction of lncRNA acting as miRNA decoys. **Table S12.** Identification of DELs acting as miRNA targets.

## Data Availability

The reference genome and genes of maize are available from RefGen_V4 (http://www.gramene.org/). The datasets generated during the current study are available in the Genome Sequence Archive (GSA) in National Genomics Data Center, China National Center for Bioinformation / Beijing Institute of Genomics, Chinese Academy of Sciences, under accession number CRA003872 that are publicly accessible at https://ngdc.cncb.ac.cn/gsa.

## Abbreviations

bHLH: Basic helix-loop helix protein
bZIP: Basic leucine zipper
CNCI: Coding-Non-Coding Index
CPC: Coding Potential Calculator
DELs: Differentially expressed lncRNAs
DEMs: Differentially expressed mRNAs
DETs: Differentially expressed transcripts
FDR: False discovery rate
FTs: Functional transcripts
GO: Gene ontology
KEGG: Kyoto Encyclopedia of Genes and Genomes
KME: Eigengene connectivity
lincRNAs: Long intergenic ncRNAs
LncRNAs: Long non-coding RNAs
mRNAs: Messenger RNAs
ORF: Open reading frame
PCA: Principal component analysis
PCC: Pearson correlation coefficient
PCs: Principal components
PCTs: Protein coding transcripts
qRT-PCR: Quantitative real-time PCR
RNA-seq: RNA sequencing
ROS: Reactive oxygen species
TFs: Transcription factors
TOM: Topological overlap measure
WGCNA: Weighted gene co-expression network analysis
