# Supplementary material for: Integrated analysis of long non-coding RNAs and mRNAs reveals the regulatory network of maize seedling root responding to salt stress
Source: BMC Genomics. 2022 Jan 13;23:50. doi: 10.1186/s12864-021-08286-7 (PMC8756644; doi:10.1186/s12864-021-08286-7)
Supplement: Supplementary file 1 — Additional file 1: Figure S1. COGs annotation for novel mRNAs. Different alphabets indicate various COGs. Detailed information of COGs can be found in the COG database (https://www.ncbi.nlm.nih.gov/research/cog/). Figure S2. qRT-PCR validation of identified lncRNAs and PCTs. Figure S3. Venn diagrams for DELs in different comparable groups. Figure S4. Determination of soft thresholding power in the WGCNA. The left panel shows the influence of soft threshold power on the scale free topological fit index; the right panel shows the influence of soft threshold power on the average connectivity. [file 12864_2021_8286_MOESM1_ESM.docx]

Integrated Analysis of Long Non-coding RNAs and mRNAs Reveals the Regulatory Network of Maize Seedling Root Responding to Salt Stress

**Authors**

Peng Liu, Yinchao Zhang, Chaoying Zou, Cong Yang, Guangtang Pan, Langlang Ma, Yaou Shen*

**Affiliations**

Key Laboratory of Biology and Genetic Improvement of Maize in Southwest Region, Maize Research Institute, Sichuan Agricultural University, Chengdu, 611130, P. R. China.

***Address for Correspondence**

Yaou Shen, Key Laboratory of Biology and Genetic Improvement of Maize in Southwest Region, Maize Research Institute, Sichuan Agricultural University, No. 211 Huimin Road, Chengdu, Sichuan, 611130, P. R. China. Email: shenyaou@sicau.edu.cn


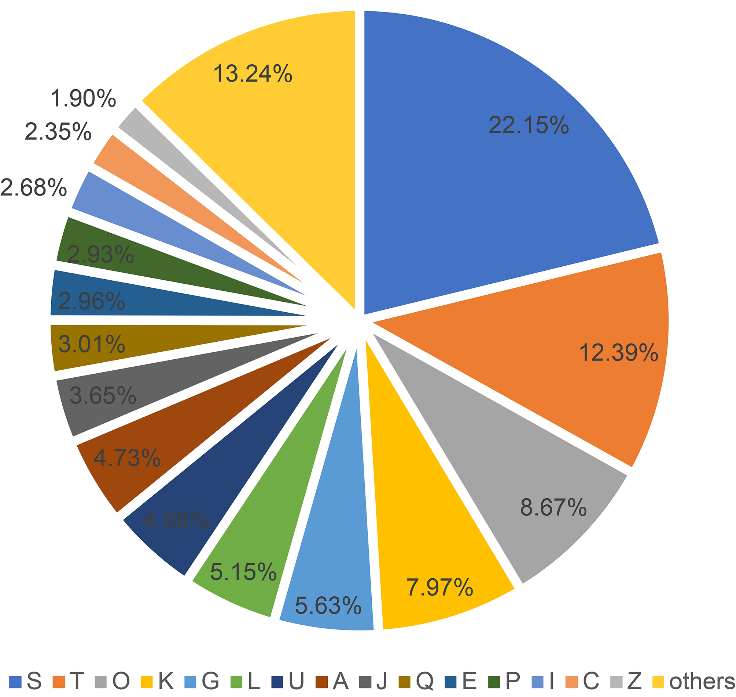


**Figure S1.** COGs annotation for novel mRNA. Different alphabets indicate various COGs. Detailed information of COGs can be found in the COG database (https://www.ncbi.nlm.nih.gov/research/cog/).


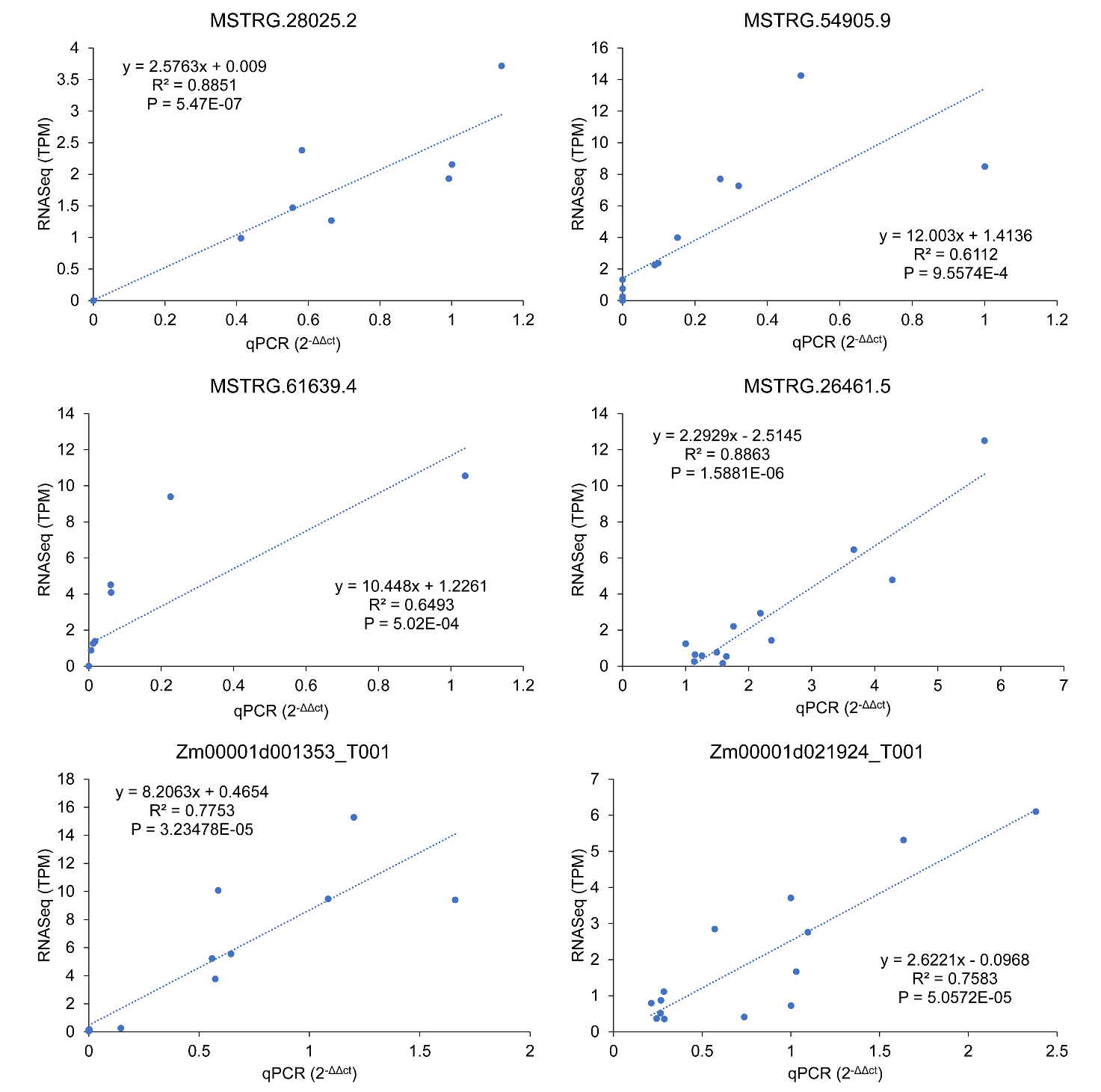
**Figure S2**. qRT-PCR validation of identified lncRNAs and PCTs.


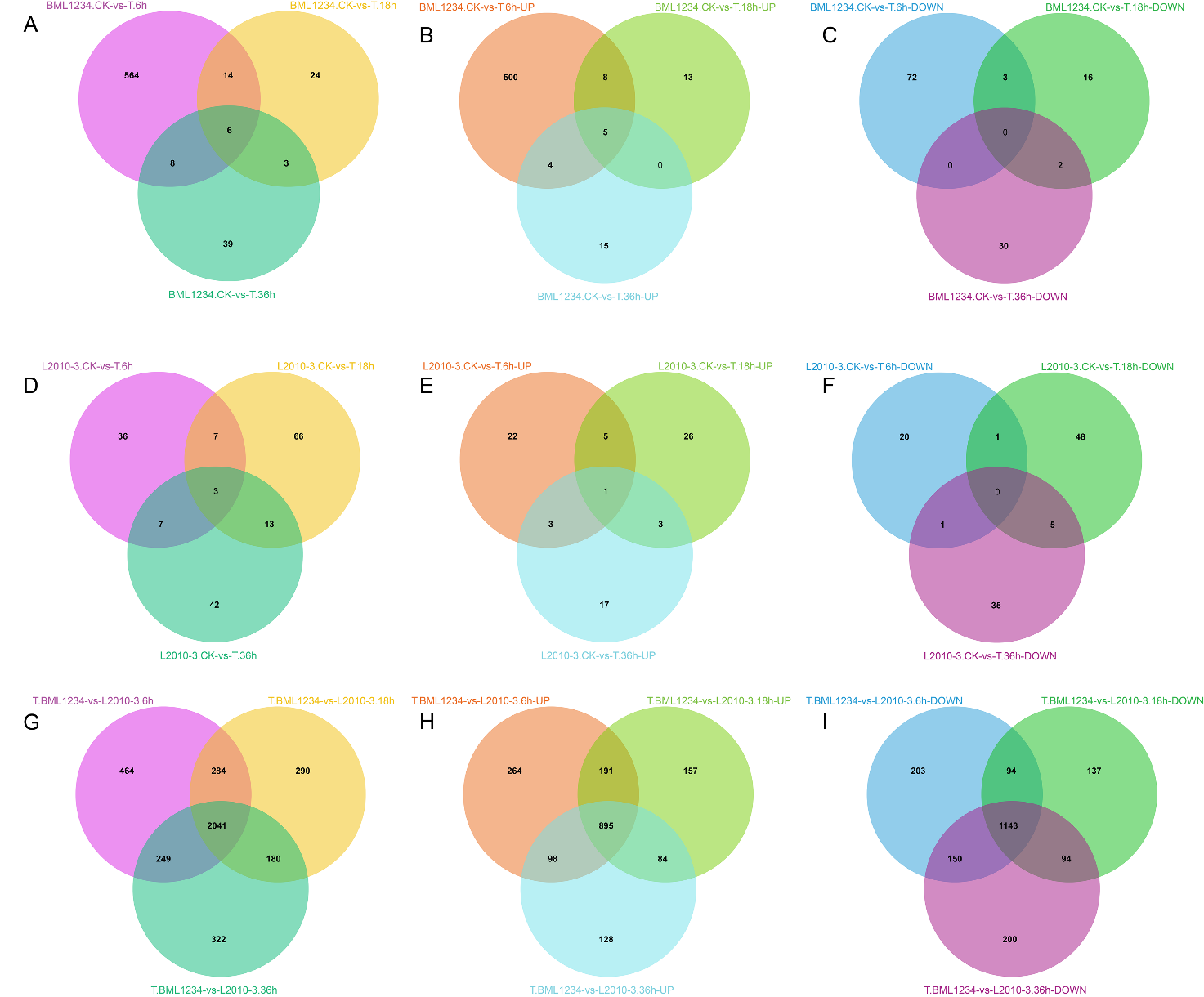


**Figure S3.** Venn diagrams for DELs in different comparable groups.


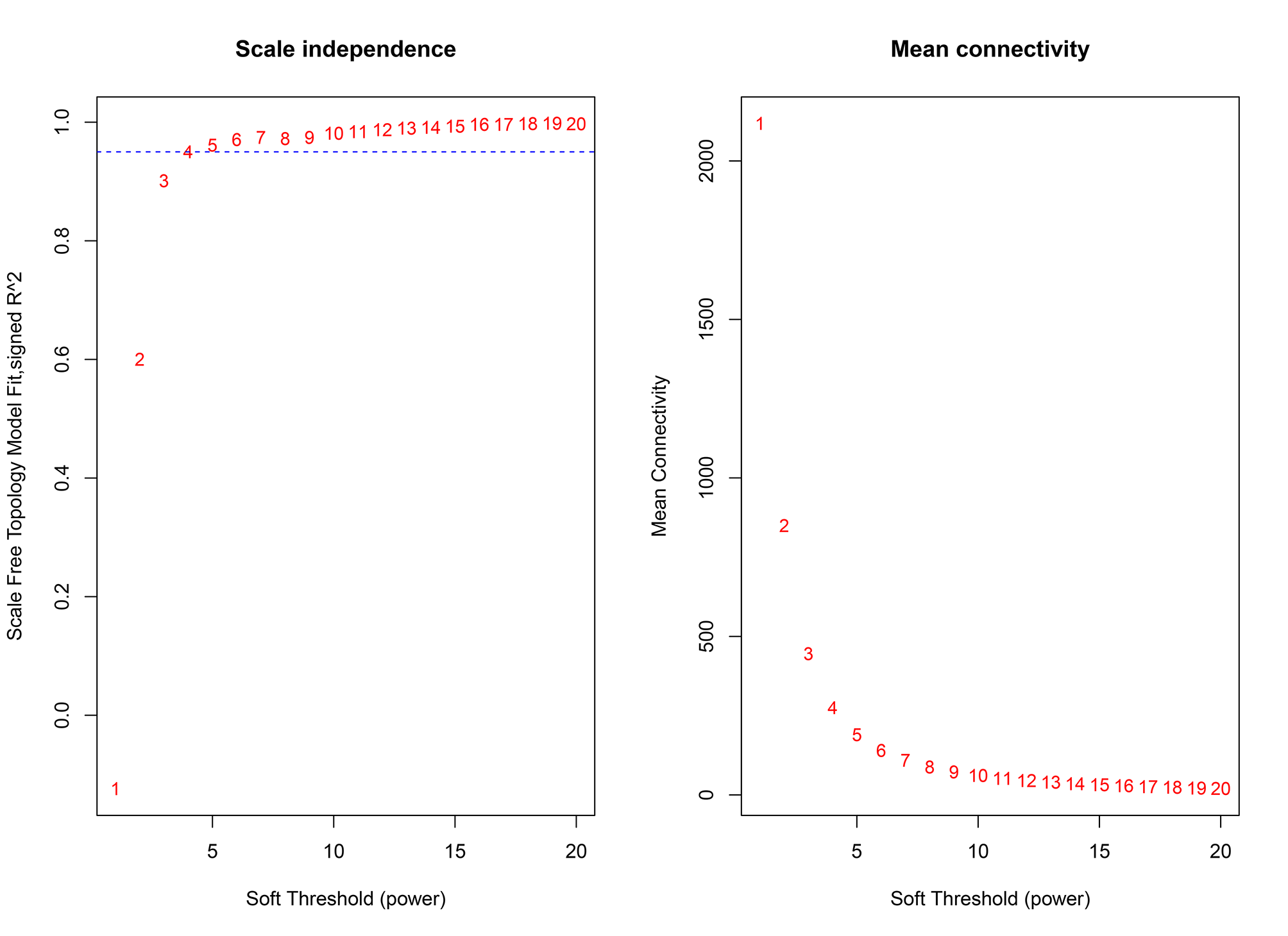


**Figure S4.** Determination of soft thresholding power in the WGCNA. The left panel shows the influence of soft threshold power on the scale free topological fit index; the right panel shows the influence of soft threshold power on the average connectivity.
